# DNA-assembled superconducting 3D nanoscale architectures

**DOI:** 10.1038/s41467-020-19439-9

**Published:** 2020-11-10

**Authors:** Lior Shani, Aaron N. Michelson, Brian Minevich, Yafit Fleger, Michael Stern, Avner Shaulov, Yosef Yeshurun, Oleg Gang

**Affiliations:** 1 Institute of Superconductivity, Department of Physics, Bar-Ilan University, 5290002 Ramat-Gan, Israel; 2 Bar-Ilan Institute of Nanotechnology and Advanced Materials (BINA), 5290002 Ramat-Gan, Israel; 3 Department of Applied Physics and Applied Mathematics, Columbia University, New York, NY 10027 USA; 4 Department of Chemical Engineering, Columbia University, New York, NY 10027 USA; 5 Quantum Nanoelectronics Laboratory, Department of Physics, Bar-Ilan University, 5290002 Ramat-Gan, Israel; 6 Center for Functional Nanomaterials, Brookhaven National Laboratory, Upton, NY 11973 USA

**Keywords:** Superconducting properties and materials, Materials for devices, Organizing materials with DNA

## Abstract

Studies of nanoscale superconducting structures have revealed various physical phenomena and led to the development of a wide range of applications. Most of these studies concentrated on one- and two-dimensional structures due to the lack of approaches for creation of fully engineered three-dimensional (3D) nanostructures. Here, we present a ‘bottom-up’ method to create 3D superconducting nanostructures with prescribed multiscale organization using DNA-based self-assembly methods. We assemble 3D DNA superlattices from octahedral DNA frames with incorporated nanoparticles, through connecting frames at their vertices, which result in cubic superlattices with a 48 nm unit cell. The superconductive superlattice is formed by converting a DNA superlattice first into highly-structured 3D silica scaffold, to turn it from a soft and liquid-environment dependent macromolecular construction into a solid structure, following by its coating with superconducting niobium (Nb). Through low-temperature electrical characterization we demonstrate that this process creates 3D arrays of Josephson junctions. This approach may be utilized in development of a variety of applications such as 3D Superconducting Quantum interference Devices (SQUIDs) for measurement of the magnetic field vector, highly sensitive Superconducting Quantum Interference Filters (SQIFs), and parametric amplifiers for quantum information systems.

## Abstract (web summary)

Fabrication of superconducting 3D nanoarchitectures, using standard nanofabrication methods, is challenging. Here, the authors demonstrate the fabrication of a nanostructured 3D superconducting array of Josephson junctions, exploiting self-assembled DNA origami lattices as a template.

## Introduction

Traditional nanofabrication methods^1^ exploiting “top-bottom” construction techniques, e.g., e-beam lithography, have been successful in producing one-dimensional (1D) and two-dimensional (2D) superconducting nanostructures, but have shown their limitation in producing three-dimensional (3D) nanostructures. In contrast, self-assembly methods offered the ability to create 3D nano-structured materials, but they were limited in ability to create prescribed 3D architectures of superconducting materials. Recently, a self-assembly of diblock polymers in gyroid phase was shown as a way to create 3D mesoporous superconductors^2^. This approach evidently demonstrates an exceptional power of self-assembly in creating 3D nano-structured superconductors, however, the diversity of possible nanostructures might be limited by the phase diagram of diblock polymers.

Over the past decades, the role of DNA in constructing nanoscale structures has been increasingly recognized^3–8^. In particular, DNA-based assembly methods present opportunities for creating engineered 3D nanoscale structures and integrate them with functional inorganic nano-components^9–12^. Recent studies demonstrate that such highly engineered object as DNA origami^13^, which emerged as a powerful technique for fabrication of complex shaped nano-objects^14–18^ and targeted nanoparticle clusters^19,20^, can be utilized for assembly of 3D lattices^21–23^. DNA origami is a process of molecular self-folding, which involves the folding of a long single strand of DNA, aided by multiple smaller “staple” strands. These staple strands bind the long strand in predetermined places, resulting in the formation of a pre-defined nanoscale structures. DNA origami nanostructures are addressable with nanometer precision, making them suitable for building precise organization of functional materials with optical, magnetic and catalytic functions^24–28^. Recently, approaches for creating designed 3D superlattices from 3D DNA origami frames and nanoparticles of different kinds were demonstrated^9,17^. This allowed to transfer advances from design of individual DNA constructs into a fabrication of architectured 3D nanostructured matter^17,21,22^ in which optical and chemical functions were revealed^17^. Parallel to the self-assembly, templating processes are needed to introduce desired material properties. For, example, DNA strands were proposed to construct the scaffold for integrated 2D molecular electronics, and to be used for patterning and other applications. Therefore, approaches for metalizing DNA and DNA origami were explored^29–32^, as well as mineralization of DNA into carbon^33^ and silicated structures^34,35^ was reported.

In this work, we show that the flexibility of the DNA-assembly can serve as a platform for both assembly of complex, pre-designed 3D periodic nanoscale architecture, and its templating with superconducting material. The 3D DNA superlattice was assembled from DNA origami frames and functionalized by coating it with superconducting Nb. The resulting structure—a 3D array of Josephson junctions—was confirmed by detailed microscopic structural and chemical mapping studies and magneto-transport measurements.

## Results

### Assembly of DNA superlattice

**Fig. 1:**
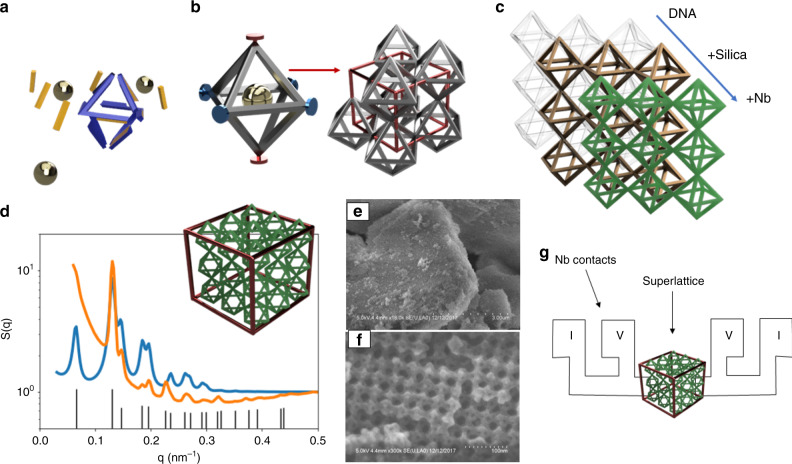
Schematics of 3D superlattice assembly from octahedra DNA frames and gold nanoparticle, and its conversion into silica and superconductive structure. **a** DNA origami octahedral frames. **b** Integration of DNA frames with gold 10 nm nanoparticles and assembly of frames into superlattice with cubic unit cell. (AuNP used here for structural characterization and are not shown in other schematics). **c** Stepwise conversion process from DNA superlattices to SiO_2_ and to Nb-coated structures. **d** Schematic of the formed simple cubic arrangement of octahedra frames. A tetragonal arrangement AuNP’s is determined by SAXS: data (orange), model (blue), indexed diffraction peaks, (001), (100), (101), (111), (002), (200) etc., are shown with vertical black lines. SEM micrographs of **e** large scale and **f** close up images of fabricated silica superlattices. **g** Schematic of low-temperature electrical measurement setup for Nb superlattice using four-point probe.

DNA origami octahedral frames (Fig. 1a) were designed using caDNAno software^36^, as previously desribed^37^. Briefly, each edge of the frames is composed of a six-helix bundle (6HB) of 28.6 nm length (84 base pairs). One single-stranded ≈2 nm DNA chain (“sticky end”) at each end of the 6HB is designed to be complementary to the DNA chain of the opposing DNA origami. In order to enhance structural characterization, 10 nm gold nanoparticles (AuNP) were inserted into the octahedron cage (golden spheres in Fig. 1b), using a DNA shell complementary to the inner strands of the frame^17^. We then designed an assembly of a simple cubic superlattice of octahedra (57 nm unit cell in an aqueous environment) with two pairs of frames designed with specific DNA strands targeting four complementary counterparts in-plane (blue) and two counterparts out-of-plane (red), as shown in Fig. 1b. This design allows placing AuNP in every alternating layer in out-plane direction, thus forming tetragonal lattice in respect to AuNP (Supplementary Fig. S1), see S4 for the lattice, frames, and AuNP design details. The separation of in-plane and out-of-plane bonds facilitates a faster in-plane growth that yields platelet type crystals, which is beneficial for Nb deposition steps and electrical characterization, as we discuss below.

The formed superlattices samples are flakes of 5–10 µm in length and 1–3 µm in thickness. Superlattice flakes were converted from a DNA material into a solid structure by sol–gel wet chemistry, growing a layer of silica on the DNA bundles. Probing the silicated structure with small angle X-ray scattering (SAXS) reveals a simple cubic arrangement of octahedron and a tetragonal arrangement of AuNP on the superlattice shown in Fig. 1d. The process of silication resulted in a shrinking of the unit cell to 48 × 48 × 48 nm^3^ as a precursor template for Nb deposition. For further details please see Supplementary Information S1. The created 3D silicated structures were further imaged using a scanning electron microscopy (SEM), as shown in the representative images in the Fig. 1e, f. The electrical properties of the Nb-coated superlattices were probed by low-temperature electrical measurements (Fig. 1g), as we discuss below.

### Formation of superconducting superlattice

**Fig. 2:**
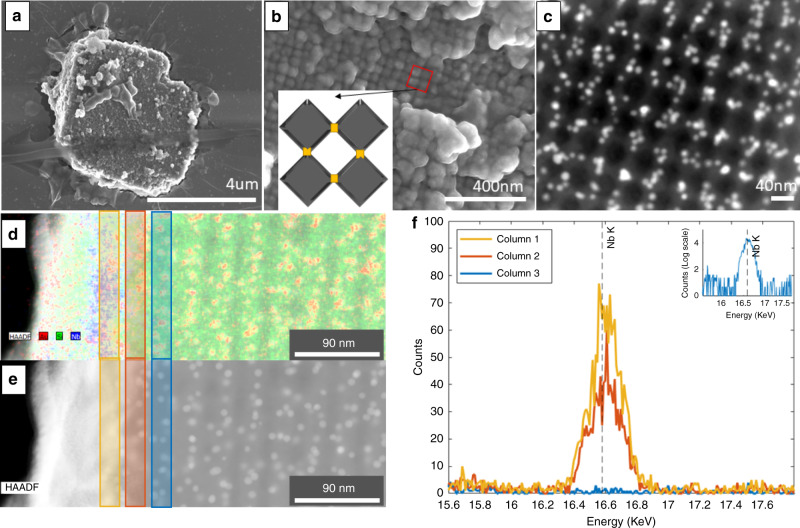
Electron microscopy characterization and element analysis of Nb-coated superlattice. **a** SEM image of the top layer of one of the Nb-coated superlattice flakes. **b** SEM of magnified region with inset figure denoting the array arrangement of S-s-S Josephson junctions with large grains grown on the octahedron (gray diamond) with small connections formed at vertices (yellow). **c** HAADF image of Nb-Superlattice representative of underlying layers of surface shown in **b**, AuNP appear white while the silica superlattice appears gray on a black background. **d** EDS map with column sums of first three layers (gold, red, and blue) of the Nb superlattice as indicated by the appearance of AuNP in red. **e** HAADF image of the same region with AuNP in white with the silica lattice in a lighter tone. **f** EDS highlighting the Nb spectrum K-edge peak at 16.58 KeV. Inset is a log scale of column 3 (blue), shown in **d**, at the same energy.

The silica converted superlattices were dispersed on a silicon chip and coated with about 10 nm thick layer of Nb, using e-beam evaporation at room temperature. The challenge in this process is to coat the structure while maintaining the lattice architecture defined by the octahedra frames. This includes avoiding sealing the voids of the structure in order to ensure penetration of the Nb through the layers and allowing coating of inner layers. At the same time, it is important to prevent penetration of Nb all the way down to the bottom of the sample, in order to avoid shorts between the electrodes in the electrical measurements. We met these requirements by controlling the evaporation rate and the temperature of the substrate, see Supplementary Information S1 for details. Figure 2a shows a SEM image of the top layer of one of the Nb-coated superlattice flakes. The Nb accumulate at the octahederal DNA structures, creating an array of relatively large Nb grains, connected by the Nb-coated sticky ends between the octahedra. The inset to Fig. 2b is a schematic view of four such grains and the weak links that connect them. As apparent from the geometry, each pair of superconducting octahedron is connected by a weak link shorter than the Nb superconducting coherence length (~40 nm), creating an ordered 3D array of *S-s-S* Josephson junctions (marked with a yellow patch in the inset), where *S* stands for the large superconducting grain and *s* for the smaller link connecting them.

In order to determine the degree of penetration of the Nb after the evaporation process, the sample was thinned from both the top and bottom, using Focused Ion Beam (Helius Dual Beam, FEI) milling, to yield a 600 nm slab of material with 10–12 layers. The top layer of this slab, i.e., an inner layer of the superlattices, was examined by Scanning transmission electron microscopy with energy dispersive spectroscopy (STEM-EDS) at the 200 keV FEI Talos microscope. Figure 2c is a STEM image of the inner layers, confirming a porous superlattice structure with AuNP tracers showing bright and with the silica-coated DNA showing a hollow framework. This structure was not filled in by Nb evaporation, and it forms a periodic array of Josephson junctions. We show in Fig. 2d, e, EDS element analysis of the outer layers of the sample, which provide information about the distribution of SiO_2_, Au and Nb in the slab. The EDS reveals the distribution of the Nb in the superlattice as being confined mostly to the top 3 pairs octahedra layers (note, AuNP are in every second layer, as discussed above, thus, a pair is empty octahedron and octahedron with AuNP), colored gold, red and blue from top to down respectively, with the blue further zoomed in inset to Fig. 2f showing the low Nb signal in the third layer. The AuNP signal as a reporter for superlattice position gives a representative number of layers into the superstructure with significant Nb deposition. By integrating column by column of superlattice EDS signal it can be determined that there was significant Nb on the first two layers of frames’ pairs and significantly smaller Nb in the third pair layer, but still detectable. Thus, the superconducting lattice is formed by about six layers of octahedra frames, with the total thickness close to 290 nm. The distribution of Nb is further presented in Supplementary Fig. S4 by considering the line scan of the sample with a longer dwell time, which shows only a few layers of Nb depositing in the top layers of the superlattice and negligible Nb in the interior of the superlattice (Supplementary Fig. S5).

### Electrical characterization

For measuring the magneto-transport properties of the Nb-coated superlattice we used laser lithography (MLA, Heidelberg Inst.) to pattern a 4-probe setup, trapping one of the coated superlattice flakes between the voltage probes. We then isolated the flake from the Nb film surrounding it, using helium ion beam (ORION NanoFab, Zeiss). (We found that the commonly used gallium ion beam deteriorates the superconducting properties of the superlattice. For details, see Supplementary Information S3). The magneto-transport characteristics of the trapped superlattice were measured using Physical Properties Measurement System (PPMS, Quantum Design).

**Fig. 3:**
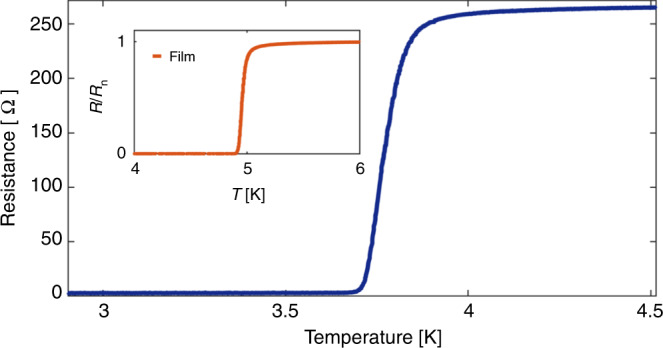
Temperature dependence of the resistance of a Nb-coated superlattice. The superconducting transition *T*_c_ ~ 3.8 K at zero field. Inset: Resistance versus temperature at zero field for the reference sample, a 10 nm thick Nb film; *T*_c_ ~ 5 K.

Figure 3 shows the temperature dependence of the resistance of a Nb-coated superlattice at zero field, demonstrating a superconducting transition temperature, *T*_c_, at ~3.8 K, lower than the transition temperature (~5 K) of the reference 10 nm thick Nb film (see inset) and the transition temperature of ~9.2 K for bulk Nb. A reduction in *T*_c_ is expected for thin layers and wires, resulting from degradation and oxidation of the superconducting material during the fabrication process^38–40^.

**Fig. 4:**
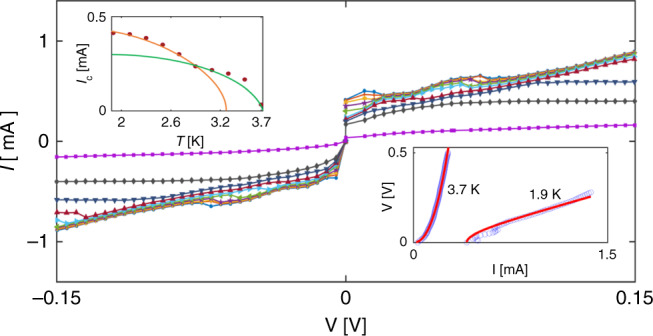
Current-voltage (*I*–*V*) characteristics of a Nb-coated superlattice. *I*–*V* characteristics for 1.9–3.7 K with Δ*T* = 0.2 *K*. Right inset: *V*–*I* characteristics (open dots) at 1.9 and 3.7 K. Solid line for the 1.9 K data is a fit to Equation (1). Solid line for the 3.7 K data is a fit to a power law, yielding *V* ∝ *I*^2.3^. Left inset: Critical current derived from the *I*–*V* curves as a function of temperature. The error in each point is estimated as 2–3%. Solid orange and green lines are guide to the eye, emphasizing the different behavior at high and low temperatures.

The current–voltage (*I*–*V*) characteristics of a Nb-coated superlattice (Fig. 4) were measured for temperatures between 1.9 and 3.7 K. The corresponding low temperature curves (1.9–2.8 K) resemble the *I*–*V* characteristics of a single Josephson junction, namely the voltage *V* = 0 for currents up to a certain temperature-dependent critical current, *I*_c_, indicated by the appearance of resistance, and then the voltage increases gradually, approaching a value *IR*_n,_ where *R*_n_ is the normal resistance. As an example, we show in the right inset to Fig. 4 a fit of the data at the lowest temperature (1.9 K) to the *I*–*V* characteristic of a Josephson junction^41^:

1 $$V = R_{\mathrm{n}}\left( {I^2 - I_{\mathrm{c}}^2} \right)^{1/2}.$$

Using Eq. (1), a reasonable fit (solid line for the 1.9 K data in the inset) is obtained with *R*_n_ = 200 Ω and *I*_c_ *=* 0.41 mA. Notably, the fit does not take into account the small variations in the voltage that are clearly observed in the *I–V* characteristics. These variations imply that the array approaches the full normal state gradually, reflecting a distribution of the Josephson critical currents in the superlattice sample. The onset of a voltage at *I*_c_ indicates that in every possible path of the current there is at least one Josephson junction, which is in the voltage state. The fact that the variations in the current are small, indicates that actually the majority of the array’s junctions in most of the current paths are in the voltage state. With increasing voltage, two competing processes determine the current; on the one hand the current follows the voltage increase and on the other hand the current drops as a result of more junctions entering the voltage state. At high enough voltage, all the junctions are in the voltage state and the measured voltage is that of the full normal state. Interestingly, at high temperatures (*T*  ≥ 3.1 K) the *I*–*V* characteristics change their nature, resembling more a power-law, which characterize systems with vortex creep^42^. As an example of this behavior, we show in the right inset a fit of a power law (solid line) to the 3.7 K data, yielding *n* = 2.3. The crossover between the low- and high-temperature behaviors is also observed in the critical current, *I*_c_, deduced from the *I*–*V* curves. As shown in the left inset, *I*_c_(*T*) exhibits discontinuity around 2.8 K. The different behavior at high and low temperatures is presumably due to the contribution of the leads to the measured resistance. While the leads do not contribute to the resistance at low temperatures, they dominate the measured resistance at temperatures close to *T*_c_ due to vortex creep.

**Fig. 5:**
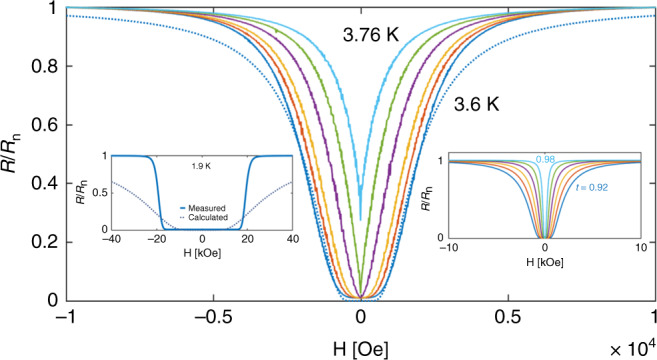
Magnetoresistance of the Nb-coated superlattice as a function of the applied magnetic field. *R* normalized to *R*_n_ = 260 Ω vs. H at temperatures 3.6, 3.62, 6.64, 3.68, 3.72, and 3.76 K. The dotted line is a fit to Equation (2) for *T* = 3.6 K. Inset: Calculated magnetoresistance based on Equation (2) for *t* = *T*/*T*_*c*_ = 0.92 to 0.98.

Qualitative different behavior at high and low temperatures is also observed in measurements of the magnetoresistance of the Nb-coated superlattice. Figure 5 shows the resistance of this sample, normalized to its normal resistance *R*_n_ ~ 260 Ω, as a function of the applied magnetic field at temperatures close to *T*_c_. A gradual increase of the resistance is observed with the field increases. An overall behavior similar to that predicted theoretically by Tinkham^43^ for the case of thermally activated flux motion, namely:

2 $$R/R_{\mathrm{n}} = (I_0[(\tilde A(1 - t)^{\frac{3}{2}})/2H])^{ - 2},$$

where *I*_0_ is the modified Bessel function, *t* = *T*/*T*_c_, $\tilde A = 80J_{{\mathrm{c}}0}$ (taking *T*_c_ = 3.8 *K*), *J*_c0_ is the critical current density at *T* = 0 and *H* = 0. The field H and the parameter $\tilde A$ are in units of G, the temperature T in Kelvin and *J*_c0_ is in units of A/cm^2^. A fit of Equation (2) to the 3.6 K data, shown by the dotted line in the figure, yields $\tilde A = 2.2 {\,} \times 10^5\,G$. We plot in the right inset the calculated *R/R*_n_ vs*. H* from Equation (2) for the same temperatures shown in the main panel. The similarity between the data and the theoretical curves is apparent. The value of $\tilde A = 2.2 {\,} \times 10^5\,G$ implies $J_{{\mathrm{c}}0}\left( 0 \right) = 2.7 {\,} \times 10^3{\mathrm{A/cm}}^2$, a reasonable value for a granular material. However, the fitting of Equation (2) to the R(H) data fails at low temperature, as demonstrated in the left inset where we show the measured data at 1.9 K together with the calculated curve using $\tilde A = 2.2 {\,} \times 10^5\,G$. Clearly, the data exhibit much steeper increase from zero to *R*_n_. This behavior is more consistent with that expected from a Josephson junction in a magnetic field^44^. The Josephson critical current, *I*_c_, is suppressed at high fields, approaching zero in the limit of the upper critical field, H_c2_. As *I*_c_ drops below the measuring current, the junction enters the voltage state and gradually approaches the normal resistance as the field continues to increase.

In summary, we demonstrated, a “bottom-up” method to create 3D superconducting superlattices (with simple cubic symmetry and 48 nm unit cell) using self-assembly of 3D DNA frames, followed by its conversion into a periodic nanoscale Nb architecture. Microscopic characterization depicts that it consists of arrays of weakly linked superconducting grains, suggesting a 3D array of Josephson junctions. Measurements of the *I*–*V* characteristics and the magnetoresistance confirm the Josephson junction behavior. This work demonstrates a conceptually different route for fabrication of complex superconducting structures that could not be prepared using conventional methods. Taking into account a tailorable platform provided by DNA-based assembly for creating engineered 3D nano-arrays^17^, the presented approach might be potentially utilized for a fabrication of arbitrary designed 3D superconducting materials with precise nano- and meso-scale organization.

## Methods

### DNA origami synthesis

DNA origami polyhedral frames were designed using caDNAno software (http://cadnano.org). Each edge of the frames is composed of a six-helix bundle (6HB). For octahedral the length of each 6HB is 28.6 nm (84 base pairs). M13mp18 DNA scaffold and DNA staple strands were mixed in a 1:5 ratio in 1x TAE buffer (40 mM Tris Acetate, 1 mM EDTA), with 12.5 mM Mg^2+^ and slowly annealed over 20 h from 90 °C to room temperature over the course of 20 h for origami formation, overall a −0.2 C/h ramp rate.

### AuNP functionalization

This method is described in [23] and reproduced here in summary. Gold NPs (10 nm) functionalized with citric acid were purchased from Ted Pella. NPs were modified with alkanethiol oligonucleotides by adding oligonucleotides to the aqueous NP solution at the mole ratio of 300:1 between DNA and NPs. After mixing for 2 h, the solution was buffered at pH 7.4 (10 mM phosphate buffer). Salt (NaCl) was added gradually to the mixture until reaching the final concentration of 0.3 M. Twelve hours later, excessive reagents were removed by centrifugation for 60 min at 15,700 r.c.f. and washed four times with 0.1 M phosphate-buffered saline buffer (0.1 M NaCl, 10 mM phosphate). The nucleotides were designed to coordinate particles to the interior of the octahedron cage.

### Superlattice formation

Four origami were synthesized to emphasize growth in-plane. Four origami were designed with specific DNA strands targeting four complementary counterparts in plane and two counterparts out-of-plane thru a second complementary origami. The sample was annealed over five days from 50 °C to room temperature (RT) at −0.2 deg/h. The designed system is modeled in Supplementary Fig. S1a, with blue, yellow, green and red signifying DNA pairs that form the specific bond.

### Silication

DNA origami superlattices were made robust by growing a layer of Silica on the DNA bundle. For conversion to inorganic silica, superlattices were centrifuged and supernatant replaced with 0.1xTAE with 10 mM Mg. Samples were brought into a cold room at 4 °C, incubated with (3-Aminopropyl)-triethoxysilane (APTES) for 30 min and then Tetraethoxysilane (TEOS) was slowly added to the lattice at a vigorous mix speed over 2 h, incubated at 4 °C for an additional 2 h, then slowly brought to RT over 24 h in a thermomixer at 1000 RPM from 10–20 °C degrees. Silicated samples were drop cast to a silicon substrate. Representative images of the sample are included in Supplementary Fig. S1b–e.

### Nb coating

The coating process begins with drop casting the superlattice structures on a 10 × 10 mm^2^ Si substrate with a native layer of oxide. The sample was dried in vacuum for 12 h and then inserted to “Plassys” e-beam evaporator and pumped for an additional 12 h to achieve optimal vacuum conditions. The sample was evaporated at a rate of 0.25 nm/s in ambient conditions to achieve film thickness of 10 nm and immersed immediately in iso-propyl alcohol (IPA) to prevent oxidation. Evaporations at low temperature produced a sheet of Nb on top of the superlattice that did not enter the cavities.

### Focused Ion Beam(FIB)/Scanning Transmission Electron Microscopy (STEM) preparation

The FIB Helios Nanolab was operated with a Gallium source to cut and mount the samples to an omniprobe grid for subsequent STEM characterization. See Supplementary Fig. S3 shows the sample in various stages of preparation.

The 200 KeV TALOS electron microscope was then used to perform EDS mapping of the structure to determine the distribution of Nb on the sample with both line scans (Supplementary Fig. S4), and maps of the interior (Supplementary Fig. S5).

### Magneto-transport measurements

We fabricated two types of 4-point probe setups, using either Ga or He ion milling, which will be described below.

### Ga ion milling

The process starts with drop casting silica superlattice on 10 × 10 mm^2^ Si chip followed by coating ~10 nm of Nb using e-beam evaporator. The structure is then lifted from the film using the FIB omni-probe to a pre-patterned 4-probe gold electrodes on Si chip, see Supplementary Fig. S6a. After connecting it to the electrodes using local Platinum deposition. The sample is cooled down in order to measure the resistance as a function of temperature. The measurements show a phase transition from normal to superconducting with zero electric resistance at ≈2 K that is drastically low compared to bulk Nb (*T*_c_ ≈ 9.2 K), see Supplementary Fig. S6b.

### He ion milling

The Nb-coated superlattice on a chip is spin coated with photoresist (AZ1518 at 4000RPM). The pattern was exposed using Mask Less Alignment (MLA) Heidelberg using 405 nm laser. Figure 1g in the manuscript show a schematic description of the pattern.

We exposed it in a negative tone, after development we get 4-point constructs on the substrate from unexposed photoresist. To form the 4-point, it is required to remove the Nb layer around the protected area, this is done using Cl2-BCl3 RIE followed by immersing in acetone at 40 °C to remove the photoresist. The final product is a 4-point with flakes of DNA between the electrodes. To isolate the flake from its surrounding substrate we use He ion milling (Orion NanoFab, Carl Zeiss), using the He-focused ion beam with 25 kV accelerating voltage and current of 1–2 pA, see Supplementary Fig. S7. In this case, *T*_c_ = 3.8 K.

## Supplementary information

Supplementary Information

Description of Additional Supplementary Files

Supplementary Dataset 1

## Data Availability

Scattering was modeled with ScatterSim available on github.com/CFN-softbio/ScatterSim. Other data can be made available upon reasonable request. Requests should be made to og2226@columbia.edu.

## References

[1] Liddle JA, Gallatin GM (2016). Nanomanufacturing: a perspective. ACS Nano.

[2] Robbins SW (2016). Block copolymer self-assembly–directed synthesis of mesoporous gyroidal superconductors. Sci. Adv..

[3] Jones MR, Seeman NC, Mirkin CA (2015). Programmable materials and the nature of the DNA bond. Science.

[4] Benson E (2015). DNA rendering of polyhedral meshes at the nanoscale. Nature.

[5] Hopkins DS, Pekker D, Goldbart PM, Bezryadin A (2005). Quantum interference device made by DNA templating of superconducting nanowires. Science.

[6] Braun E, Keren K (2004). From DNA to transistors. Adv. Phys..

[7] Stulz E (2012). DNA Architectonics: towards the Next Generation of Bio‐inspired Materials. Chem.–A Eur. J..

[8] Seeman NC, Gang O (2017). Three-dimensional molecular and nanoparticle crystallization by DNA nanotechnology. Mrs Bull..

[9] Kahn J, Minevich B, Gang O (2020). Three-dimensional DNA-programmable nanoparticle superlattices. Curr. Opin. Biotechnol..

[10] Zhao Z, Jacovetty EL, Liu Y, Yan H (2011). Encapsulation of gold nanoparticles in a DNA origami cage. Angew. Chem. Int. Ed. Engl..

[11] Sun DZ, Gang O (2013). DNA-functionalized quantum dots: fabrication, structural, and physicochemical properties. Langmuir.

[12] Zhang YG, Lu F, Yager KG, van der Lelie D, Gang O (2013). A general strategy for the DNA-mediated self-assembly of functional nanoparticles into heterogeneous systems. Nat. Nanotechnol..

[13] Rothemund PW (2006). Folding DNA to create nanoscale shapes and patterns. Nature.

[14] Wang P, Meyer TA, Pan V, Dutta PK, Ke Y (2017). The beauty and utility of DNA origami. Chem.

[15] Tian C (2017). Supra-nanoparticle functional assemblies through programmable stacking. ACS Nano.

[16] Gerling T, Wagenbauer KF, Neuner AM, Dietz H (2015). Dynamic DNA devices and assemblies formed by shape-complementary, non–base pairing 3D components. Science.

[17] Tian Y (2020). Ordered three-dimensional nanomaterials using DNA-prescribed and valence-controlled material voxels. Nat. Mater..

[18] Han D (2011). DNA origami with complex curvatures in three-dimensional space. Science.

[19] Sun S (2020). Valence-programmable nanoparticle architectures. Nat. Commun..

[20] Roller E-M (2015). DNA-assembled nanoparticle rings exhibit electric and magnetic resonances at visible frequencies. Nano Lett..

[21] Liu W (2016). Diamond family of nanoparticle superlattices. Science.

[22] Tian Y (2016). Lattice engineering through nanoparticle–DNA frameworks. Nat. Mater..

[23] Zhang T (2018). 3D DNA origami crystals. Adv. Mater..

[24] Wang, P. F. et al Magnetic plasmon networks programmed by molecular self-assembly. *Adv. Mater.* **31**, 10.1002/adma.201901364 (2019). 10.1002/adma.201901364 31148269

[25] Zhang H (2020). Polarized single-particle quantum dot emitters through programmable cluster assembly. ACS Nano.

[26] Zhao Z (2016). Nanocaged enzymes with enhanced catalytic activity and increased stability against protease digestion. Nat. Commun..

[27] Urban MJ (2016). Plasmonic toroidal metamolecules assembled by DNA origami. J. Am. Chem. Soc..

[28] Meyer TA, Zhang C, Bao G, Ke Y (2020). Programmable assembly of iron oxide nanoparticles using DNA origami. Nano Lett..

[29] Schreiber R (2011). DNA origami-templated growth of arbitrarily shaped metal nanoparticles. Small.

[30] Jia S (2019). rigami patterning with non-canonical DNA-based metallization reactions. Nat. Commun..

[31] Mertig M, Colombi Ciacchi L, Seidel R, Pompe W, De Vita A (2002). DNA as a selective metallization template. Nano Lett..

[32] Keren K, Berman RS, Braun E (2004). Patterned DNA metallization by sequence-specific localization of a reducing agent. Nano Lett..

[33] Zhou F (2016). Programmably shaped carbon nanostructure from shape-conserving carbonization of DNA. ACS Nano.

[34] Nguyen L, Döblinger M, Liedl T, Heuer-Jungemann A (2019). DNA-origami-templated silica growth by sol–gel chemistry. Angew. Chem. Int. Ed..

[35] Liu X (2018). Complex silica composite nanomaterials templated with DNA origami. Nature.

[36] Douglas S (2009). Self-assembly of DNA into nanoscale three-dimensional shapes. Nature.

[37] Tian Y (2015). Prescribed nanoparticle cluster architectures and low-dimensional arrays built using octahedral DNA origami frames. Nat. Nanotechnol..

[38] Park S, Geballe T (1985). Tc depression in thin Nb films. Phys. B+ C..

[39] Kim H, Jamali S, Rogachev A (2012). Superconductor-insulator transition in long moge nanowires. Phys. Rev. Lett..

[40] Pereiro J, Saerbeck T, Schuller IK (2015). Effect of increasing disorder on superconductivity of Mo/Nb superlattices. Superconductor Sci. Technol..

[41] Tinkham, M. *Introduction to Superconductivity*. 2nd edn, 202–211, (Dover Publications Inc., 2004).

[42] Yeshurun Y, Malozemoff A, Shaulov A (1996). Magnetic relaxation in high-temperature superconductors. Rev. Mod. Phys..

[43] Tinkham M (1988). Resistive transition of high-temperature superconductors. Phys. Rev. Lett..

[44] Zhilyaev I (2013). Step-like dependence of the magnetoresistance of a Josephson structure. J. Surf. Investig. X-ray, Synchrotron Neutron Tech..

