# Supplementary material for: DNA-assembled superconducting 3D nanoscale architectures
Source: Nat Commun. 2020 Nov 10;11:5697. doi: 10.1038/s41467-020-19439-9 (PMC7656258; doi:10.1038/s41467-020-19439-9)
Supplement: Supplementary file 1 — Supplementary Information [file 41467_2020_19439_MOESM1_ESM.pdf]

Supplementary Information

DNA-assembled superconducting 3D nanoscale architectures

L. Shani *et al*.

S1. Sample preparation

DNA origami synthesis

DNA origami polyhedral frames were designed using caDNAno software (http://cadnano.org). Each edge of the frames is composed of a six-helix bundle (6HB). For octahedral the length of each 6HB is 28.6 nm (84 base pairs). M13mp18 DNA scaffold and DNA staple strands were mixed in a 1:5 ratio in 1 x TAE buffer (40mM Tris Acetate, 1mM EDTA), with 12.5 mM Mg^{2+} and slowly annealed over 20hrs from 90°C to room temperature over the course of 20 hrs for origami formation, overall a -0.2 C/hr ramp rate.

AuNP functionalization

Gold NPs (10 nm) functionalized with citric acid were purchased from Ted Pella. NPs were modified with alkanethiol oligonucleotides by adding oligonucleotides to the aqueous NP solution at the mole ratio of 300:1 between DNA and NPs. After mixing for 2 h, the solution was buffered at pH 7.4 (10 mM phosphate buffer). Salt (NaCl) was added gradually to the mixture until reaching the final concentration of 0.3 M. Twelve hours later, excessive reagents were removed by centrifugation for 60 min at 15,700 r.c.f. and washed four times with 0.1 M PBS buffer (0.1 M NaCl, 10 mM phosphate). The nucleotides were designed to coordinate particles to the interior of the octahedron cage.

Superlattice Formation

Four origami were synthesized to emphasize growth in-plane, 4 origami were designed with specific DNA strands targeting 4 complementary counterparts in plane and 2 counterparts out-of-plane thru a second complementary origami. The sample was annealed over 5 days from 50°C to RT at -0.2 deg/hr. The designed system is modeled in figure S1a, with blue, yellow, green and red signifying DNA pairs that form the specific bond.

Silication

DNA origami superlattices were made robust by growing a layer of Silica on the DNA bundle. For conversion to inorganic silica, superlattices were centrifuged and supernatant replaced with 0.1xTAE with 10mM Mg. Samples were brought into a cold room at 4°C, incubated with (3-Aminopropyl)-triethoxysilane (APTES) for 30 minutes and then Tetraethoxysilane (TEOS) was slowly added to the lattice at a vigorous mix speed over 2 hours, incubated at 4°C for an additional 2 hours, then slowly brought to RT over 24 hours in a thermomixer at 1000 RPM from 10-20 ^{o}C degrees. Silicated samples were drop cast to a silicon substrate. Representative images of the sample are included in Figure S1(b-e).

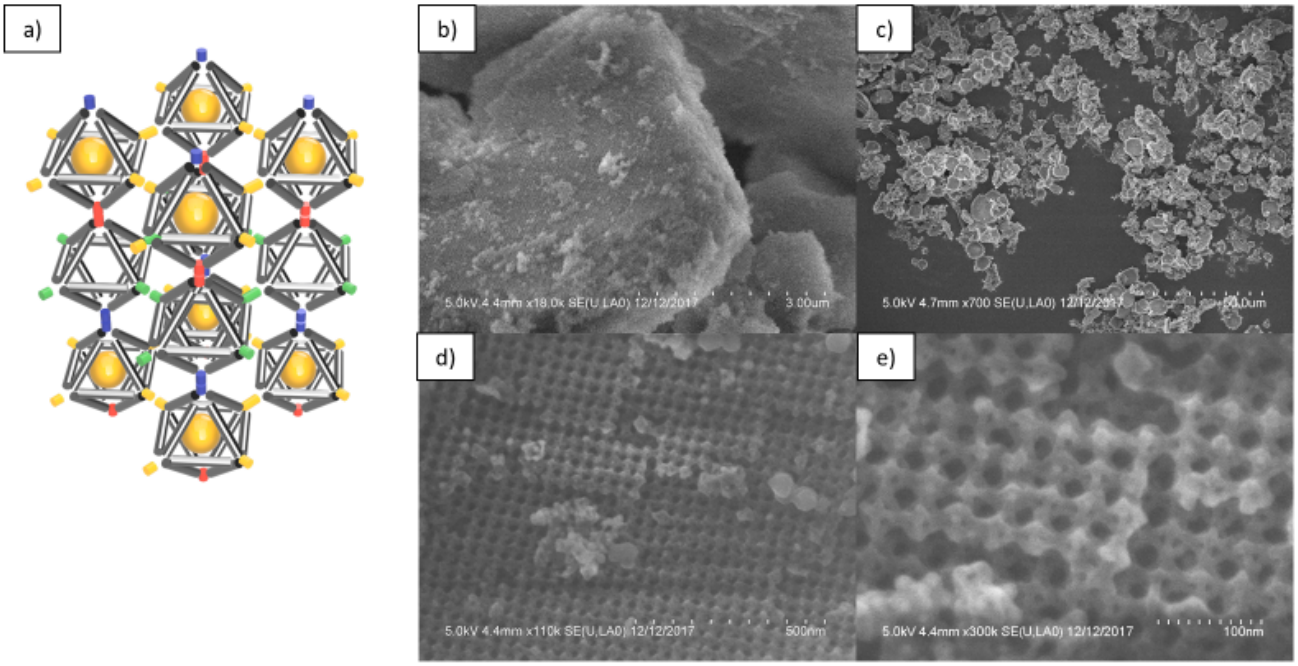

Figure S1. a) DNA-Origami Superlattice model with octahedra frames modeled with colored vertexes representative of DNA binding. The four in plane bonds compared to two out of plane bonds on each individual octahedron act to exaggerate growth out of plane. See also S4 for the detailed design. b-e) Representative images of the superlattices after silication where octahedron and gold can be visualized forming platelet type superlattice particles with approximately a 5-10μm edge length.

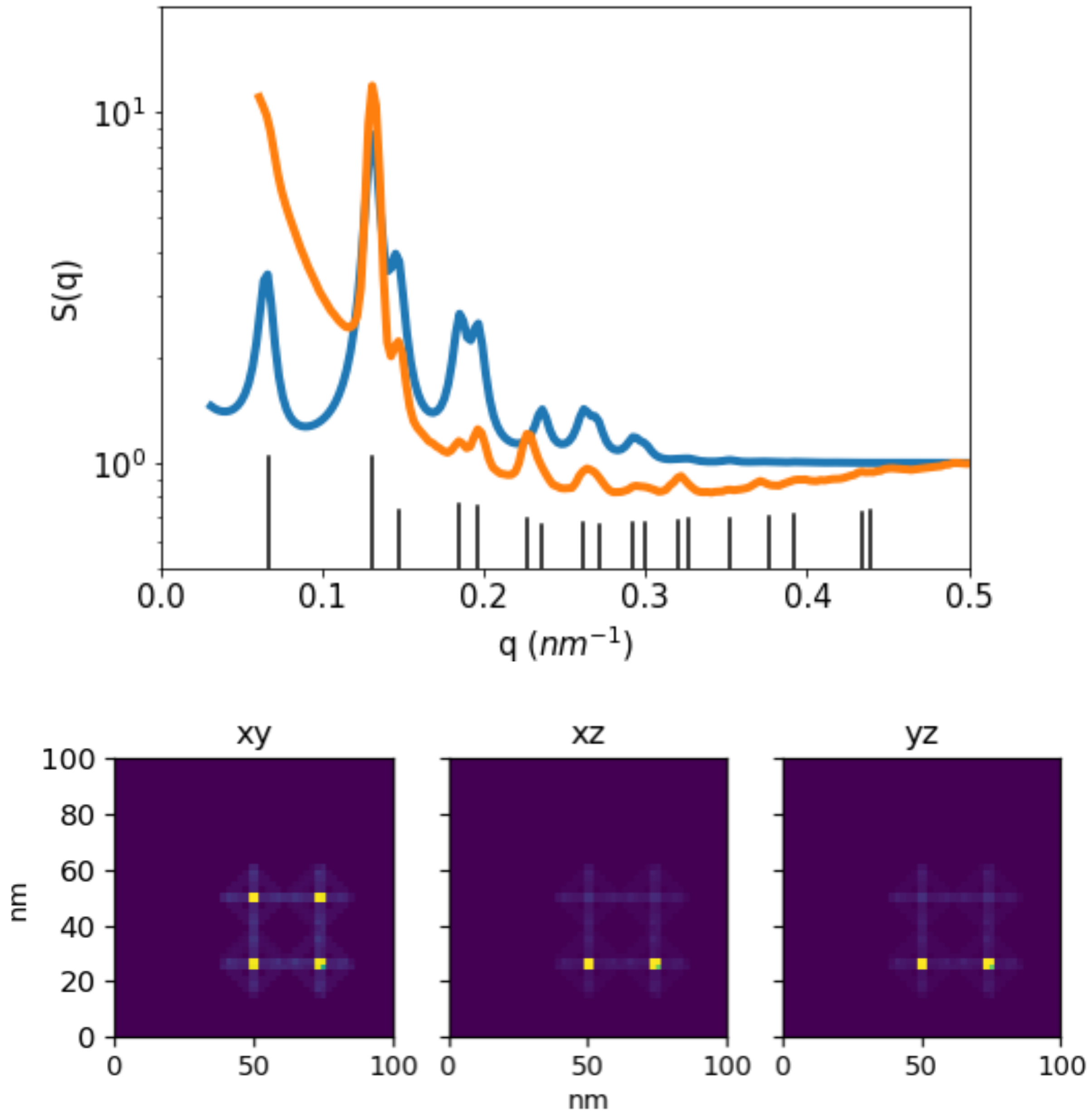

Figure S2 Small angle scattering of superlattices in aqueous solution (yellow curve). Model scattering of a tetragonal lattice made up of octahedron and gold 5nm spheres (blue). To model the superlattice we use ScatteSim[1]. From left to right the following reflections are shown in black: (001), (100), (101), (111), (002), (200), (012), (120), (112), (211), (022), (220), (003), (122), (221), (300), (013), (310).The 100 set of planes representative of octahedron center to center distances is 48nm. XY, XZ and YZ slices of the model scatterers are represented below the 1D saxs curve.

Nb Coating

The coating process begins with drop casting the superlattice structures on a 10x10 mm^2 Si substrate with a native layer of oxide. The sample was dried in vacuum for 12 hours and then inserted to 'Plassys' e-beam evaporator and pumped for an additional 12 hours to achieve optimal vacuum conditions. The sample was evaporated at a rate of $0.25\ nm/sec$ in ambient conditions to achieve film thickness of 10 nm and immersed immediately in iso-propyl alcohol (IPA) to prevent oxidation. Evaporations at low temperature produced a sheet of Nb on top of the superlattice that did not enter the cavities.

S2. Microscopic characterization

Sample preparation for TEM imaging

The FIB Helios Nanolab was operated with a Gallium source to cut and mount the samples to an omniprobe grid for subsequent TEM characterization. The sample was mounted vertically due to precipitous break of the sample diagonally across the square profile. The sample was thinned from both the top and bottom to yield a 600nm slab of material nominally containing between 10-12 layers. Figure S3 shows the sample in various stages of preparation. SEM imaging of the sample (top-down view and side view) show progressive thinning where Au NP are visible or invisible (due to being blocked by intervening empty layers of DNA origami).

The 200 KeV TALOS electron microscope was then used to perform EDS mapping of the structure to determine the distribution of Nb on the sample with both line scans Figure S4, and maps of the interior Figure S5.

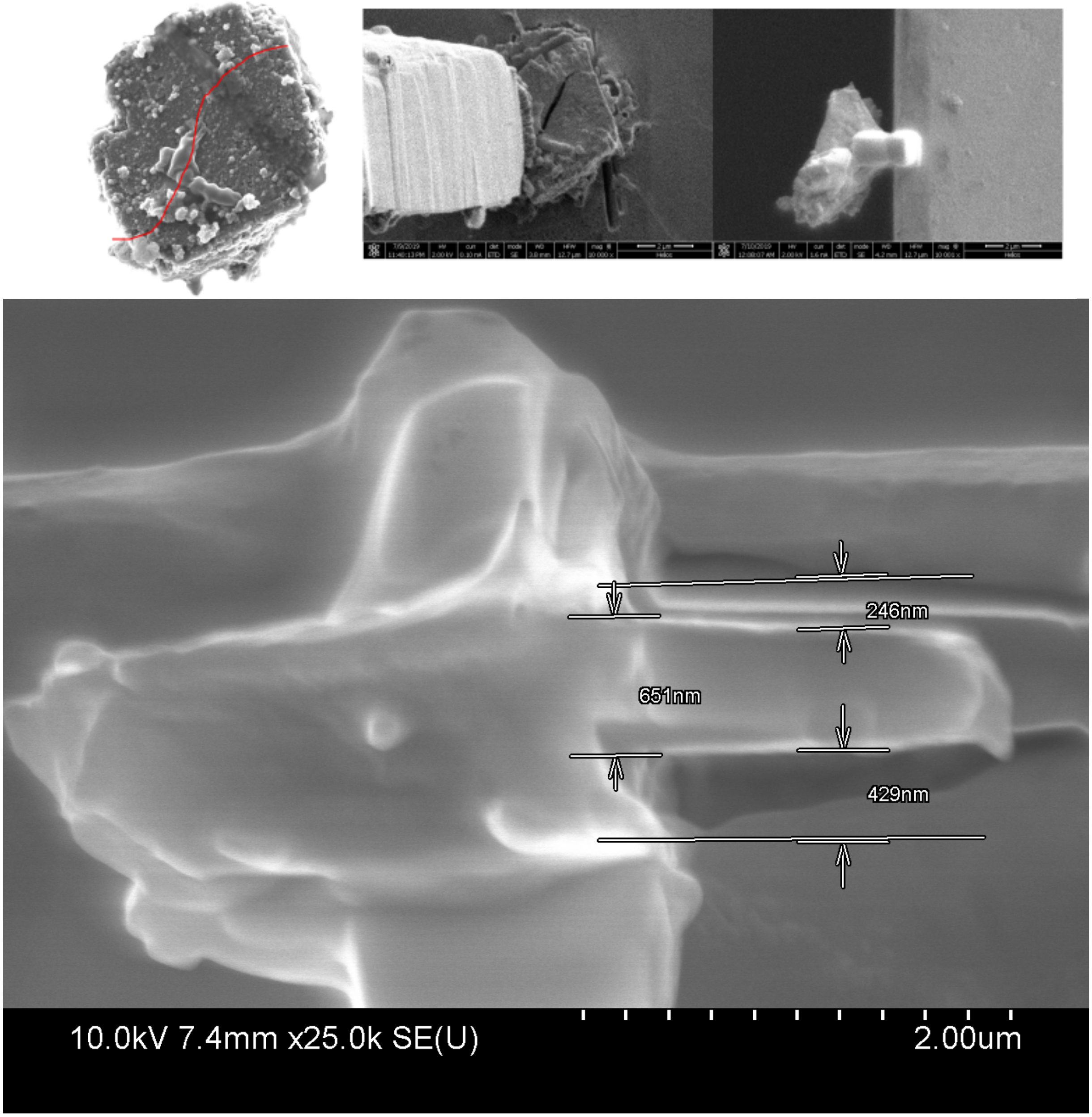

Figure S3 Nb Sample Fib/SEM preparations, shown in red is the region that partially broke when picked up by the omniprobe. The section was mounted to the B section of an omniprobe liftout grid. The sample was then thinned (shown from top down view) with ~650nm of the sample remaining.

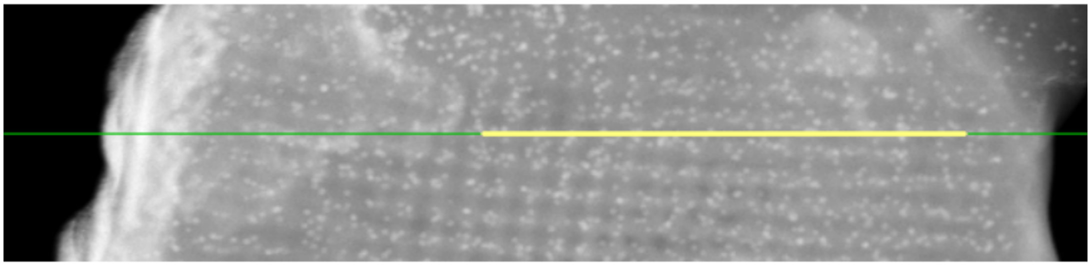

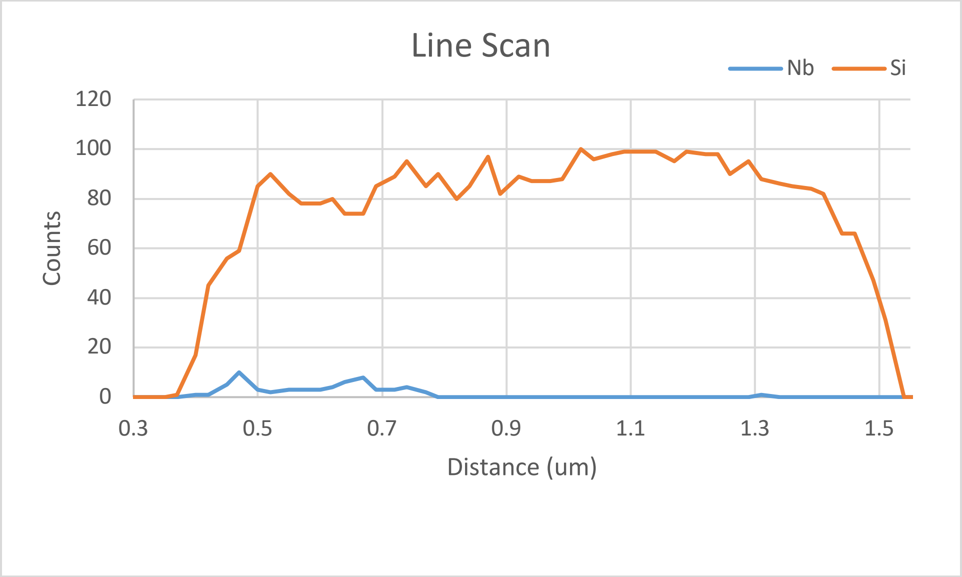

Figure S4. Line scan of the sample following Silicon and Niobium counts. A thickness variation on the left side of the sample is likely from slight curtaining of the surface of the sample.

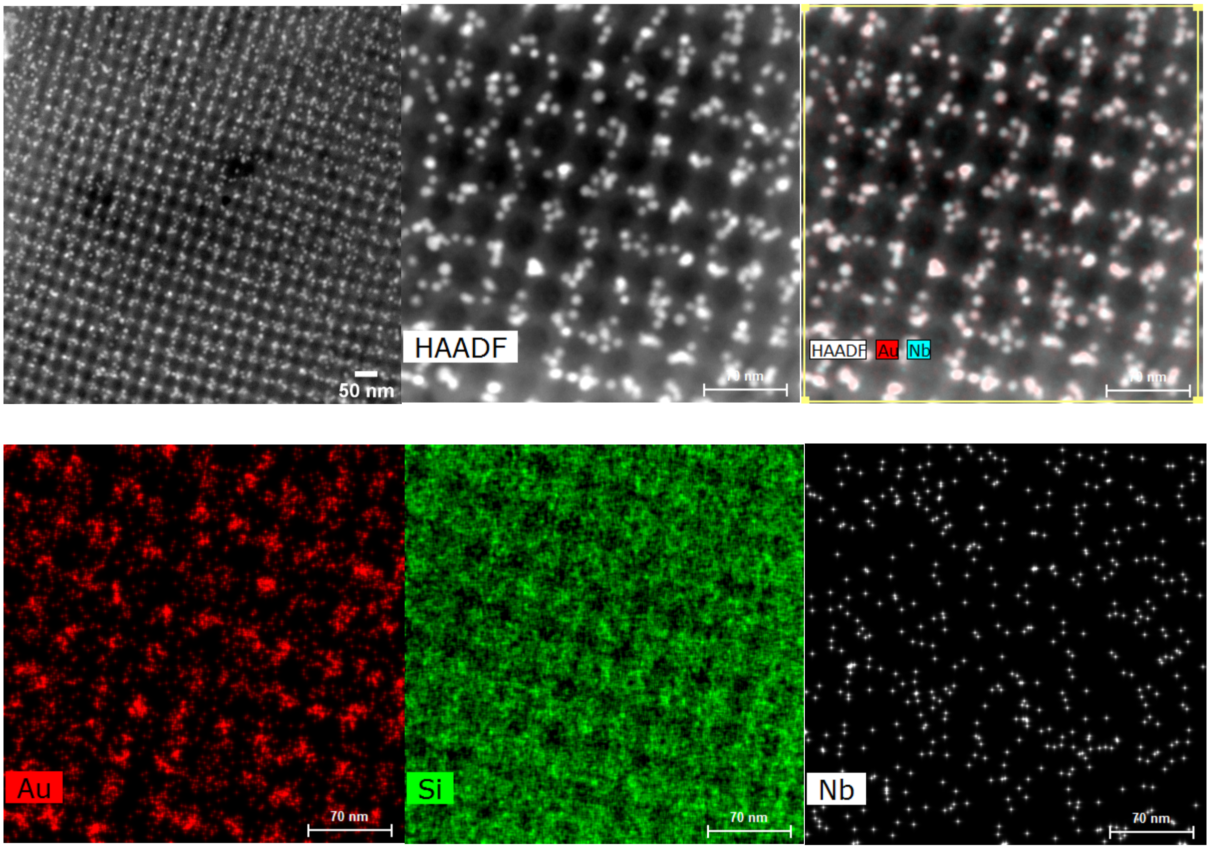

Figure S5. HAADF and EDS Map of the superlattice. Large area magnification of the sample along with EDS-HAADF images of the sample with Au, Si, Nb counts displayed along with an overlay map of Nb and AU on the HAADF image. This image shows that very little Nb penetrates into the interior of the sample and is mostly confined to the surface layers of the superlattice as shown in the main text.

S3. Magneto-transport measurements

4-probe setup

We have fabricated two types of setups, using either Ga or He ion milling, described below.

Sample preparation using Ga ion milling:

The process starts with drop casting silica superlattice on 10X10 mm^2 Si chip followed by coating ~10 nm of Nb using e-beam evaporator. The structure is then lifted from the film using the FIB omni-probe to a pre-patterned 4-probe gold electrodes on Si chip, see Figure S6a. After connecting it to the electrodes using local Platinum deposition. The sample is cooled down in order to measure the resistance as a function of temperature. The measurements show a phase transition from normal to superconducting with zero electric resistance at $\approx 2\ K$ that is drastically low compared to bulk Nb ($T_c \approx 9.2\ K$), see figure S6b.

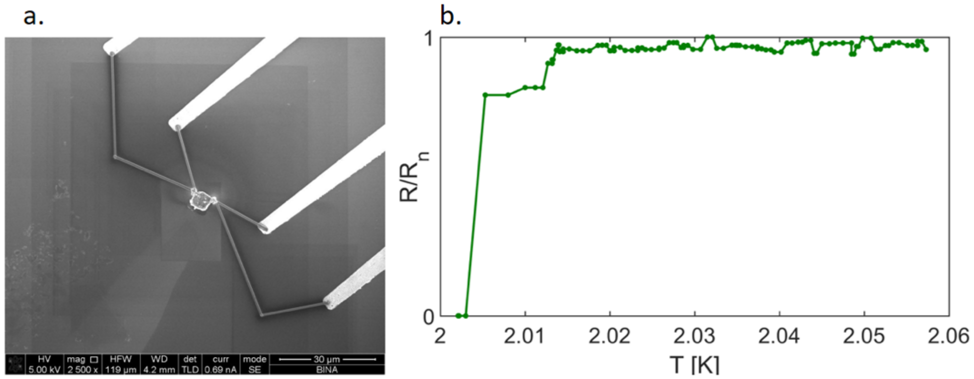

Figure S6. Electrical characterization. (a) SEM image of the Nb coated superlattice setup (b) Resistance as a function of temperature, $T_c \approx 2\ K$.

Sample preparation using He ion milling:

The Nb coated superlattice on a chip is spin coated with photoresist (AZ1518 at 4000RPM). The pattern was exposed using Mask Less Alignment (MLA) Heidelberg using 405 nm laser. Figure 1g in the manuscript show a schematic description of the pattern.

We exposed it in a negative tone, after development we get 4-point constructs on the substrate from unexposed photoresist. To form the 4-point, it is required to remove the Nb layer around the protected area, this is done using Cl2-BCl3 RIE followed by immersing in acetone at 40 C to remove the photoresist. The final product is a 4-point with flakes of DNA between the electrodes. To isolate the flake from its surrounding substrate we use He ion milling (Orion NanoFab, Carl Zeiss), using the He focused ion beam with 25 kV accelerating voltage and current of 1-2 pA, see Figure S7. In this case, T_c = 3.8 K.

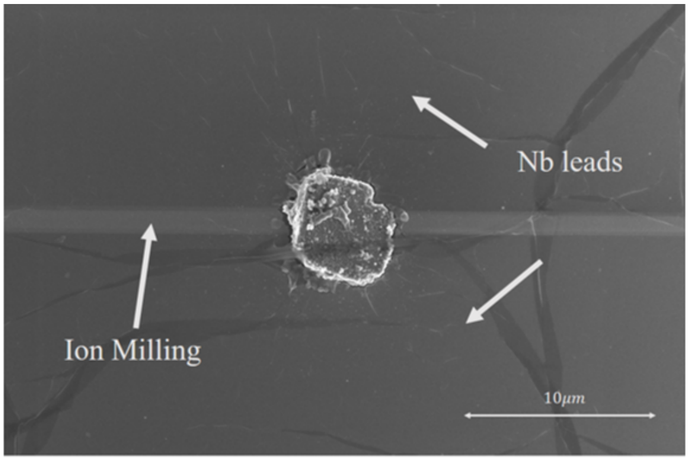

Figure S7. SEM image of the Nb coated superlattice. Arrow on the left show the area that was removed using ion milling. Arrows on the right show the Nb leads for the external measurement instrument.

S4. Design of superlattice and DNA origami frames

Lattice design

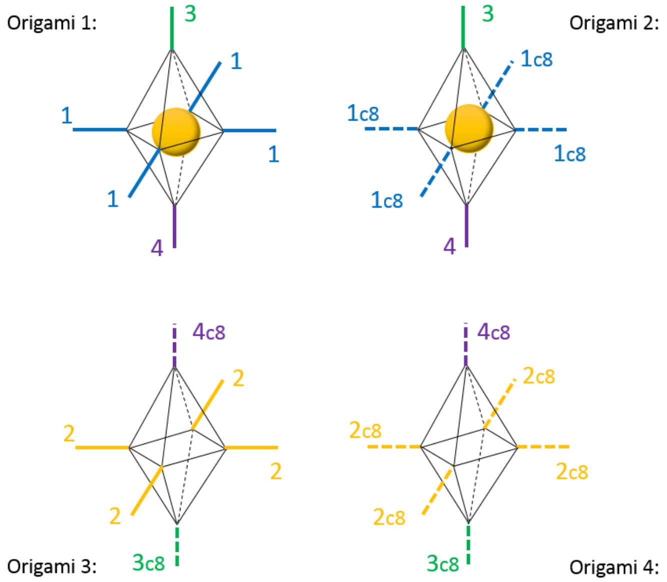

Figure S8. Design of the lattice from 2 pairs of octahedra origami frames. One pair in top layer (1, 2) host AuNP and another pair (3, 4) of octahedra frames is empty. Similar colors represent complementarity of sticky ends (shown as solid and dashed lines)

DNA Origami Designs

Supplementary Data Tables are provided in the online version of this document, see Supplementary Data 1.xlsx

Supplementary References

1. G., Zhang, Y., Lu, F. & Gang, O. Periodic lattices of arbitrary nano-objects: modeling and applications for self-assembled systems. *Journal of Applied Crystallography* 47, 118-129, doi:doi:10.1107/S160057671302832X (2014).
