# Supplementary material for: DNA-assembled superconducting 3D nanoscale architectures
Source: Nat Commun. 2020 Nov 10;11:5697. doi: 10.1038/s41467-020-19439-9 (PMC7656258; doi:10.1038/s41467-020-19439-9)
Supplement: Supplementary file 2 — Description of Additional Supplementary Files [file 41467_2020_19439_MOESM2_ESM.pdf]

Description of Additional Supplementary Files

File Name: Supplementary Data 1
Description: DNA sequences for DNA origami designs
